# On the energy scale involved in the metal to insulator transition of quadruple perovskite EuCu_3_Fe_4_O_12_: infrared spectroscopy and ab-initio calculations

**DOI:** 10.1038/srep28624

**Published:** 2016-06-27

**Authors:** B. Brière, A. Kalinko, I. Yamada, P. Roy, J. B. Brubach, R. Sopracase, M. Zaghrioui, V. Ta Phuoc

**Affiliations:** 1 GREMAN, CNRS UMR 7347-CEA, Université F. Rabelais, UFR Sciences, Parc de Grandmont, Tours, 37200, France; 2 Synchrotron Soleil, Université Paris-Saclay, L’Orme des Merisiers, 91190 Saint-Aubin, France; 3 Nanoscience and Nanotechnology Research Center, Osaka Prefecture University, Sakai, Osaka Japan

## Abstract

Optical measurements were carried out by infrared spectroscopy on AA′_3_B_4_O_12_ A-site ordered quadruple perovskite EuCu_3_Fe_4_O_12_ (microscopic sample) as function of temperature. At 240 K (=T_*MI*_), EuCu_3_Fe_4_O_12_ undergoes a very abrupt metal to insulator transition, a paramagnetic to antiferromagnetic transition and an isostructural transformation with an abrupt large volume expansion. Above T_*MI*_, optical conductivity reveals a bad metal behavior and below T_*MI*_, an insulating phase with an optical gap of 125 meV is observed. As temperature is decreased, a large and abrupt spectral weight transfer toward an energy scale larger than 1 eV is detected. Concurrently, electronic structure calculations for both high and low temperature phases were compared to the optical conductivity results giving a precise pattern of the transition. Density of states and computed optical conductivity analysis identified Cu_3*dxy*_, Fe_3*d*_ and O_2*p*_ orbitals as principal actors of the spectral weight transfer. The present work constitutes a first step to shed light on EuCu_3_Fe_4_O_12_ electronic properties with optical measurements and ab-initio calculations.

Investigation of A-site ordered double perovskites AA′_3_B_4_O_12_ have some appeal to the scientific community as they exhibit unexpected properties owing to their unique structures and great variety of composition. Unlike the A site of simple perovskite oxides ABO_3_, which is usually occupied by alkali, alkaline earth, or rare earth cations, the A′ site in AA′_3_B_4_O_12_ enables an accommodation of transition-metal ions, such as Cu^2+^ and Mn^3+^, which are Jahn–Teller active. Therefore, A′-A′ and A′-B interactions may give rise to novel physical properties in addition to the usual B-B interactions. Colossal magnetoresistance under weak field appears in ACu_3_Mn_4_O_12_ (A = Ca, La or Bi)^1^, heavy fermions behavior is found in ACu_3_Ru_4_O_12_^2^, Mott physics is at the play in CaCu_3_Co_4_O_12_^3^,^4^ and CaCu_3_Ti_4_O_12_ exhibits a giant dielectric constant over a wide temperature range^5^,^6^. In addition, it has recently been reported that CaCu_3_Fe_4_O_12_ shows an excellent catalytic activity for oxygen evolution reaction and its stability is enhanced by covalent bonding network between Cu–O–Fe bonds^7^.

Among A-site ordered quadruple perovskites, lanthanides composed LnCu_3_Fe_4_O_12_ (noted LnCFO in the following) present a strong interest insofar as they undergo rare valence state transitions accompanied by intriguing functional properties. Metal-insulator transition (MIT) and negative thermal expansion (NTE)^8^ are part of them. LnCFO crystallizes in a quadruple perovskite Im-3 structure ordered on the A-site of AA′_3_B_4_O_12_ with a coordination number of 12, 6 and 4 for Ln, Fe and Cu respectively. As shown in Fig. 1, the cubic geometry is filled with four FeO_6_ octahedra and CuO_4_ square planar complexes. According to the size of the Lanthanide^9^, two families of LnCFO are brought out with both their own electronic and magnetic properties. On the one hand, small size rare earth ion compounds (Ln = Dy, Ho, Er, Tm, Yb and Lu) exhibit an iron charge-disproportionation 8Fe^3.75+^ → 5Fe^3+^ + 3Fe^5+^ accompanied by a paramagnetic metal to a ferrimagnetic semiconductor transition around 255 K^10^. On the other hand, large size rare earth ions family (Ln = La, Pr, Nd, Pm, Sm, Eu, Gd and Tb) undergoes the following intermetallic charge transfer 3Cu^2+^ + 4Fe^3.75+^ → 3Cu^3+^ + 4Fe^3+^ leading to a paramagnetic-metal to an AFM-insulator phase transition between 240 K and 350 K^9^. These properties result from the transition between the high valence state of Cu^3+^ at the square-coordinated A sites and the Fe^3.75+^ at the octahedron-coordinated B sites. The charge transfer occurs under high pressure conditions for many materials^11^ but in LnCFO it is triggered by a slight change of temperature promising potential room temperature applications. For instance, the temperature transition is T_*MI*_ = 310 K in SmCFO. La_*x*_Ln_1−*x*_Cu_3_Fe_4_O_12_ substituted systems could allow a modulation of the MIT temperature and LaCu_3_Fe_4(1−*x*)_Mn_4(*x*)_O_12_ doped compounds is an example of a controlled NTE^12^. LnCFO with smaller Ln ions are subjected to volume change at temperatures that provide a good opportunity to control simultaneously both physical properties and thermal expansion and allow the development of thermal sensors^13^,^14^. This paves the way for many potential technological applications with composite and single-phase compounds^15^. EuCu_3_Fe_4_O_12_ (ECFO) is very representative of large size LnCFO and its optical properties have never been measured yet. It exhibits the earlier cited intermetallic charge transfer between Cu and Fe at 240 K traduced by a MIT, a paramagnetic to antiferromagnetic transition and an isostructural volume change^8^,^13^,^16^,^17^. On cooling, all the metal-oxygen bonds lengths change significantly. The Cu-O bonds shrink simultaneously with the charge transfer between Cu_3*dxy*_ and Fe_3*d*_ via O_2*p*_ orbitals and the Fe-O bonds expand. Although many theoretical and computational studies have been recently devoted to describe the electronic structure and the mechanism of the MIT in LnCFO^9^,^16^,^18^,^19^,^20^, experimental investigations of the electronic properties across the MIT are still missing.

In this paper, we report on low-energy electrodynamics of ECFO investigated by infrared spectroscopy. We found that ECFO undergoes an abrupt metal to insulator transition (MIT) at 240 K associated with a change of optical properties over an energy scale larger than 1 eV. The comparison with DFT calculations shows that, despite the large amount of energy involved in the MIT, ECFO is a moderately correlated system. Furthermore, both Fe_3*d*_ and Cu_3*d*_ bands are strongly modified at the transition, in relation with the charge transfer and the magnetic transition.

## Results and Discussion

### Optical measurements

**Figure 2:**
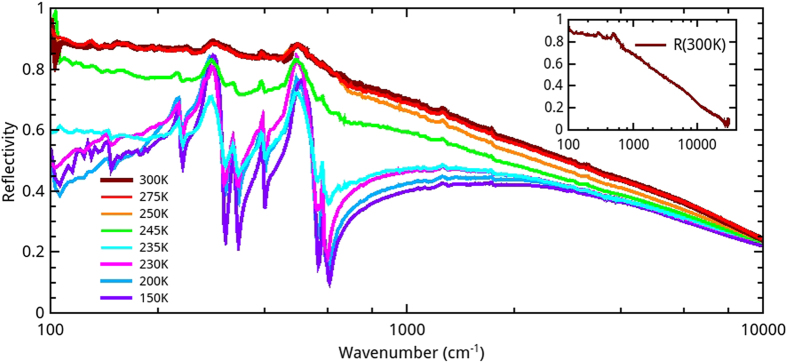
Infrared reflectivity of EuCu_3_Fe_4_O_12_ measured between 100 cm^−1^ and 10 000 cm^−1^ for various temperatures. Inset: reflectivity at room temperature up to 30 000 cm^−1^.

Reflectivity spectra of ECFO, R(*ω*), are shown for different temperatures from room temperature to around the MIT temperature in Fig. 2. For 300 K, the reflectance exhibits a metallic response, slowly decreasing from 0.9 to 0.15 over the measured frequency range. However, no clear plasma edge is observed. Such a behavior is often found in correlated transition metal oxides^21^,^22^,^23^,^24^,^25^. As the temperature is decreased down to 250 K, the spectra do not show any qualitative change. By contrast, the reflectivity is rapidly reduced between 245 K and 235 K in both far infrared and mid infrared ranges. Below 230 K, spectra display an insulating behavior and several phonon modes are detected. Seven of the 12 infrared active modes predicted by group theory can be observed.

**Figure 3:**
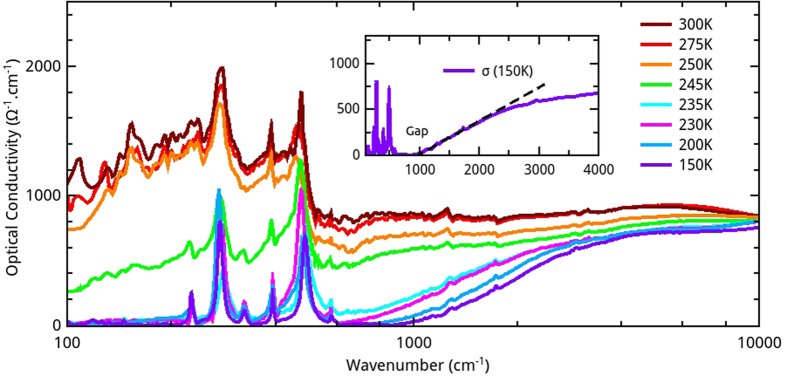
Optical conductivity spectra of EuCu_3_Fe_4_O_12_ at various temperature. Inset: The optical gap energy is determined by a linear extrapolation at the inflection point of the onset of *σ*(*ω*) (dashed line).

**Figure 4:**
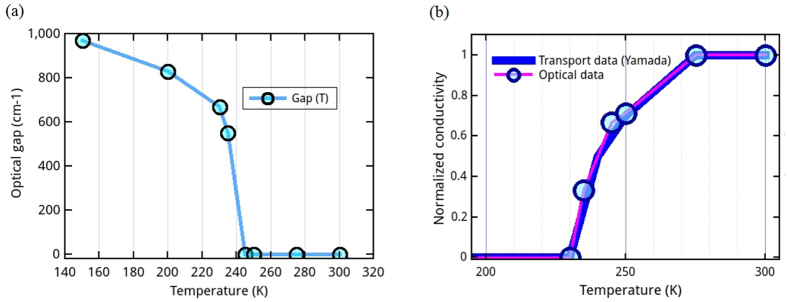
(**a**) Temperature dependence of the optical gap of ECFO. (**b**) Temperature dependent ECFO normalized conductivity deduced from optical data (open circle) and transport data (blue line)^37^. Normalization is done at 300 K.

The optical conductivity *σ*(*ω*) deduced from reflectivity spectra by Variation Dielectric Function technique (VDF)^26^,^27^,^28^ is shown in Fig. 3. In the metallic paramagnetic phase (T > 240 K), the optical conductivity is nearly flat and slightly decreases at low frequency to reach approximately 1 000 Ω^−1^cm^−1^ at 100 cm^−1^. It clearly deviates from usual Drude model since it exhibits a broad bump at 200 cm^−1^. This could be due to either localization by disorder^29^,^30^,^31^, electronic correlations^32^,^33^ or strong electron-lattice coupling (polarons)^29^,^34^,^35^. With decreasing temperature, the optical conductivity dramatically drops three order of magnitude at low frequency, and exhibits an insulating behavior, with an optical gap energy Δ ≈ 125 meV and a broad mid infrared band. Moreover, note that the optical conductivity is larger in the metallic phase than in the insulating phase up to 9 000 cm^−1^. The optical gap energy is determined by a linear extrapolation at the inflection point of the onset of *σ*(*ω*)^36^ (inset of Fig. 3). The electronic gaps extracted from optical conductivity spectra are reported in Fig. 4a. The very abrupt transition clearly deviates from a BCS-like behavior. Note that the gap value in the insulating phase (Δ ≈ 125 meV) is slightly smaller than previous DFT calculations of other LnCu_3_Fe_4_O_12_ that predict Δ between 300 and 800 meV^16^,^18^,^20^. As shown in Fig. 4b, the temperature dependence of the normalized conductivity taken at 100 cm^−1^ (the lowest measured frequency), and the one obtained from transport measurements^37^ are in very good agreement.

Note that we found a quite large discrepancy between the conductivity obtained by transport (of order of 1 Ω^−1^cm^−1^) and the value of the 300 K optical conductivity at 100 cm^−1^ (1000 Ω^−1^cm^−1^). However, since the sample is a very small polycristal, such a discrepancy can easily be due to the determination of geometrical parameters (cross section, distance between electrodes), grain boundaries and low density of the sample. In order to estimate the typical energy scale involved in the MIT mechanism, the optical conductivity spectral weight (SW) was calculated from the optical conductivity:


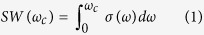


**Figure 5:**
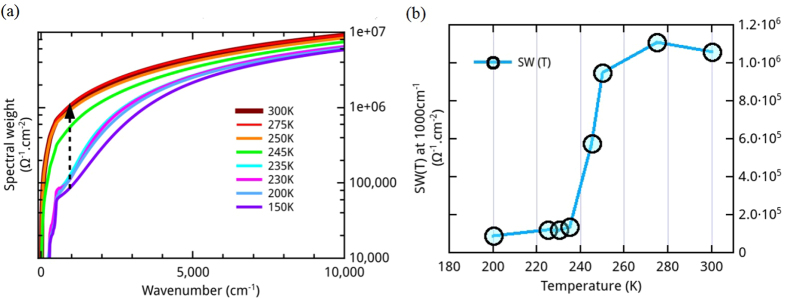
(**a**) Spectral weight calculated between 100 and 10 000 cm^−1^. The black doted arrow on SW indicates for which energy SW(T) has been determined. (**b**) SW(T) plotted at 1 000 cm^−1^ (≈ energy of the gap).

SW is related to the charge carrier density contributing to the optical properties up to a cutoff energy *ω*_*c*_. As shown in Fig. 5a, the spectral weight is not recovered up to 10 000 cm^−1^ implying that electronic properties of ECFO are modified over an unusually large spectral range at the MIT. In addition, the temperature dependence of the SW calculated (Fig. 5b) at *ω*_*c*_ ≈ 1 000 cm^−1^ (i.e. order of the gap energy) clearly shows a step-like behavior.

### Ab-initio calculations

**Figure 6:**
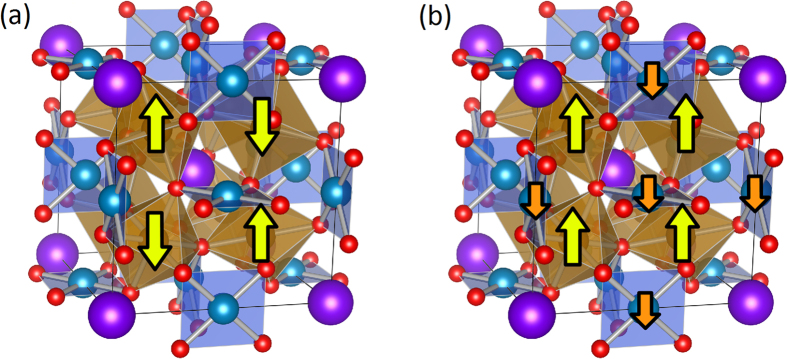
The magnetic orders considered in ECFO. (**a**) AFM: the nearest-neighbor Fe moments are aligned antiparallel to each other and Cu has no magnetic moment. (**b**) FiM: all the Fe moments are parallel and the Cu moments are antiparallel to them.

In order to get a better insight of the electronic structure and to identify low energy optical excitations, DFT + U calculations were performed. The calculation has been carried out for both the insulating and the metallic phases by using ECFO crystallographic data for 100 K and 300 K. Low temperature phase (LT) was taken anti-ferromagnetic G-type on Fe lattice, as shown in Fig. 6a, in accordance with experimental x-ray diffraction data^38^.

In order to reproduce the order of magnitude of the experimental gap, we choose U = 1.5 eV. Such a value of U is not uncommon since it usually ranges between 2 eV and 7 eV in most transition metal compounds^39^,^40^,^41^,^42^. Note that for U < 1.5 eV, DFT + U predicts a metallic ground state in disagreement with experiments. In addition, values of U > 1.5 eV do not drastically affect the physics^16^,^18^,^20^. A more detailed investigation of U parameter has been performed by Isoyama *et al*.^18^ on the LaCFO compound.

**Figure 7:**
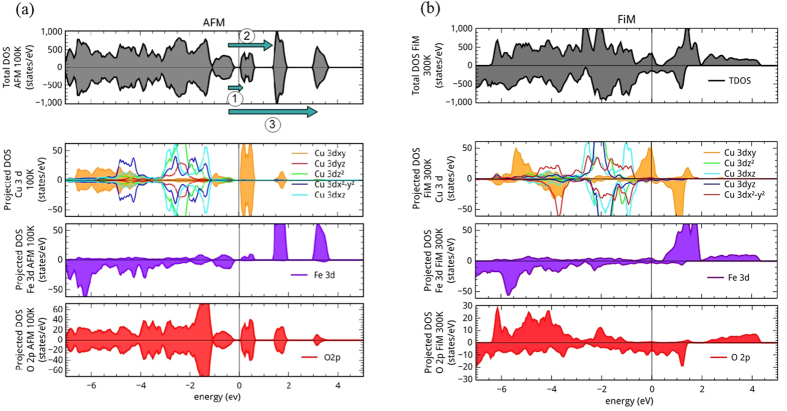
Total and Projected Density of State (spin up and spin down) calculated by DFT + U for both LT (AFM) (**a**) and HT (FiM) (**b**) phases. The three arrows shown on the AFM TDOS correspond to the three excitations picked up on the AFM DFT optical conductivity (Fig. 8).

ECFO Total Density of State (TDOS) and site Projected DOS (PDOS) are displayed in Fig. 7a,b. As expected, the TDOS of the AFM phase shows an insulating state with a gap of 250 meV that is in reasonable agreement with Δ_*exp*_ ≈ 125 meV. TDOS is constituted of a wide band of energy from −7 eV to −0.2 eV and three bands above the Fermi level, 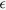
_*f*_, up to 3.75 eV. The PDOS (Fig. 7a) allows to identify a narrow spin polarized Fe_3*d*_ orbital occupancy at 1.8 eV and a strong hybridization of Cu_3*d*_ and O_2*p*_ states around 0.2 eV. Note that the Fe_3*d*_ spin state is in good agreement with neutron diffraction data^38^; the calculated Fe magnetic moment (integrated inside the Fe muffin-tin sphere) M_*Fe*_^*calc*^ = 3.97 *μ*_*B*_ is very close to the experimental one M_*Fe*_^*exp*^ = 4.03 *μ*_*B*_. The calculated Cu magnetic moment is zero in accordance with neutron diffraction data^38^. It is worth noting that PDOS of Fe_3*d*_ orbitals are all the same whereas Cu_3*dxy*_ differs from other Cu_3*d*_. More precisely, beyond 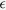
_*f*_, Cu DOS is essentially composed of Cu_3*dxy*_ orbitals state as seen in Fig. 7a and noticed by Hongping *et al*. in the study of LaCFO^16^. So Cu_3*dxy*_ orbitals hold a central role in the valence transition.

In order to describe the ECFO paramagnetic high temperature phase (HT), we used a ferrimagnetic structure FiM (as drawn in Fig. 6b) since paramagnetic structure is difficult to set up in DFT calculations. Several studies^17^,^20^ used a FiM order insofar as the physics of the metallic phase of LnCFO is quite well captured. TDOS and PDOS of the HT phase are displayed in Fig. 7b. The expected metallic state is well reproduced since 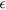
_*f*_ is occupied by a wide valence band from −7 eV to 4.2 eV. The PDOS shows that Cu_3*d*_, Fe_3*d*_ and O_2*p*_ orbitals are hybridized around the Fermi level to constitute a wide spread band driving the metallic state. Also for this phase, Cu_3*dxy*_ is the only orbital that differs from other Cu_3*d*_ orbitals by crossing 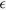
_*f*_. This supports the fact that Cu_3*dxy*_ is involved in the low frequency (<600 cm^−1^) behavior of the HT optical conductivity (Fig. 3). Moreover, Cu ions now wear a non zero magnetic moment M_*Cu*_^*calc*^ = +0.32 *μ*_*B*_ that is coupled antiparallel to the iron one of M_*Fe*_^*calc*^ = −3.60 *μ*_*B*_.

The observed calculated changes between LT and HT phases illustrate the charge transfer between Cu_3*dxy*_ site orbital and Fe_3*d*_ one via O_2*p*_ orbital hybridization. In addition, the modifications of DOS over an energy scale larger than 1.5 eV (the lowest value of U that reproduces the gap) show that the energy scale involved in the charge transfer is larger than 1.5 eV. This is roughly consistent with our spectral weight study (Fig. 5) which indicates that the mechanisms involved in the MIT occur beyond 1 eV.

**Figure 8:**
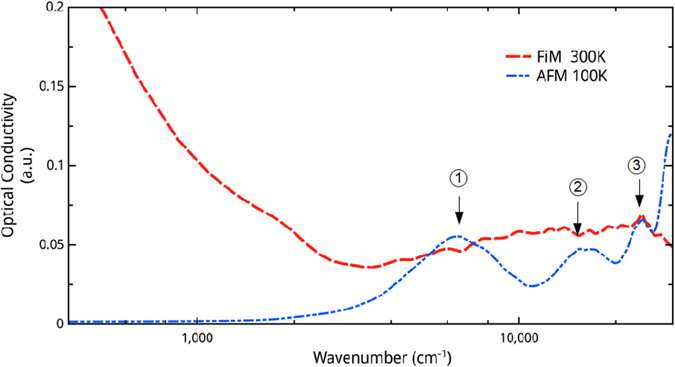
Optical conductivity calculated (*σ*_*RPA*_) by Random Phase Approximation in both high (red) and low (blue) temperature phase between 100 cm^−1^ and 30 000 cm^−1^. The three numbered arrows correspond to the three transitions noted on the AFM TDOS (Fig. 7(a)).

For the purpose of the comparison with the experiment, we have calculated the optical conductivity by Random Phase Approximation (RPA) using our DFT electronic structure calculations. Although RPA does not take into account many body effects, it is expected to roughly capture the physics. LT and HT calculated optical conductivities (*σ*_*RPA*_) are presented in Fig. 8. It is worth noting that *σ*_*RPA*_ of the LT phase exhibits an electronic gap of 250 meV (2 000 cm^−1^). This is the same order, or even larger than our experimental gap of 125 meV and previous LnCFO DFT studies^16^,^18^,^20^. The first bump at 6 000 cm^−1^ on the low temperature *σ*_*RPA*_ (blue curve) is assigned to the LT optical conductivity excitation detected at 5 000 cm^−1^ (Fig. 3). Two more excitations are predicted at 10 500 cm^−1^ and 25 000 cm^−1^. For the HT phase (metallic) *σ*_*RPA*_ presents a clear Drude-like peak below 2 000 cm^−1^ that differs from the low frequency behavior detected by spectroscopy for the HT phase (Fig. 3). Such a difference is not very surprising since DFT calculations do not incorporate temperature effects, strong electronic correlations or localization by disorder as discussed above. Nevertheless, the shape and the relative level of the calculated conductivities, are compatible with a spectral weight displacement over a large energy scale since HT conductivity is higher than the LT conductivity from 0 cm^−1^ (0 eV) to 26 000 cm^−1^ (3.2 eV). The behavior of *σ* beyond this range is necessarily in favor of the spectral weight recovering since the charge carrier density is conserved.

## Conclusion

Based on reflectivity measurements and first principle calculations, we reported the first infrared spectroscopy study of the A-site ordered quadruple perovskite EuCu_3_Fe_4_O_12_ highlighting the opening of a 125 meV gap below 240 K with an abrupt metal to insulator transition. The charge transfer involves an energy scale larger than U = 1.5 eV that reflects the presence of electronic correlations in the material. The DFT + U study has been managed with a surprising small value of U (1.5 eV). We identified the mechanism responsible for the MIT as being the redistribution of density of states from Fe_3*d*_ orbitals to Cu_3*dxy*_ with the hybridization to O_2*p*_ orbitals. The next step will consist in driving the MIT by physical pressure for others LnCFO in order to disentangle the role of magnetism in the transition.

## Methods

ECFO polycrystalline samples were synthesized under high pressure in a high pressure cell (15 GPa) and high temperature (1000 K)^37^. This technique severely restricts the volume of the sample to approximately millimeter size. The sample after synthesis has low density and is crumbly. Thus, a clean flat surface of 120 *μ*m diameter was polished up to optical grade 0.25 *μ*m. SXRD and Fe Mossbauer spectroscopy confirmed the composition of the samples and the valence state of both high and low temperature phases being EuCu^+2^_3_Fe^+3,75^_4_O_12_ and EuCu^3+^_3_Fe^3+^_4_O_12_ with a lattice constant of 7.33641(8) Å for 300 K and 7.37306(8) Å for 100 K.

Near normal incidence reflectivity spectra were measured between 100 cm^−1^ and 30 000 cm^−1^, from 150 K to 300 K. Low temperature measurements on such a small sample were obtained by using a homemade high-vacuum microscope including a x15 Schwarzchild objective connected to a BRUKER IFS 66v/S Fourier-transform spectrometer. After the initial measurement, the sample was coated with a 150 nm aluminium film and remeasured in the previous temperature range. These additional data were used as reference to calculate the reflectivity in order to take into account light scattering on the surface of the sample and possible misalignement induced by heating. Note that, due to the small size of the sample, diffraction limit is reached around 100 cm^−1^. Above 10 000 cm^−1^, light scattering due to surface roughness prevents to obtain a good signal to noise ratio. Thus, although both the shape and level of the reflectivity were reasonable according to infrared measurements and high energy reflectivity obtained from x-ray atomic scattering functions as proposed by D. B. Tanner^43^, no clear conclusion can be drawn on the temperature dependence above 10 000 cm^−1^. As a consequence, temperature dependent infrared data have been merged with the room temperature UV data. Optical conductivity spectra were obtained consistently by Variation Dielectric Function analysis (VDF) and Kramers-Kronig (KK) analysis. In order to proceed the KK analysis, reflectivity spectra were extrapolated at low frequency with Hagens-Rubens formula in the metallic phase and a constant value in the insulating phase, and by using high frequency extrapolation based on x-ray atomic scattering functions technique^43^. Spin-polarized first-principles DFT calculations have been carried out using the full-potential augmented plane-wave method with local orbitals (FP-APW + lo) as implemented in the ELK code. The generalized gradient approximation (GGA) using Perdew–Burke–Ernzerhof (PBE) parametrization was employed for the exchange-correlation energy potential. The Brillouin zone sampling was done using an uniformly spaced k-grid of 6 × 6 × 6. To avoid complexity, 4f states of europium atoms are considered as core states^44^, since they are not expected to be involved in low energy electronic properties. The structural parameters were taken from experimental X-ray crystallographic data at 100 K and 300 K, to describe low temperature (LT) and high temperature (HT) phases respectively. The broadening used to compare with the data is 0.25 eV^45^. In order to take into account the strong correlations for both Fe and Cu 3d electrons, DFT + U method was used to add the on-site Hubbard U_*eff*_ = U−J term. Hereinabove, the term U_*eff*_ is denoted just as U for simplicity. The dielectric function was computed in the Random Phase Approximation (RPA).

## Additional Information

**How to cite this article**: Brière, B. *et al*. On the energy scale involved in the metal to insulator transition of quadruple perovskite EuCu_3_Fe_4_O_12_: infrared spectroscopy and ab-initio calculations. *Sci. Rep.* **6**, 28624; doi: 10.1038/srep28624 (2016).

## Figures and Tables

**Figure 1:**
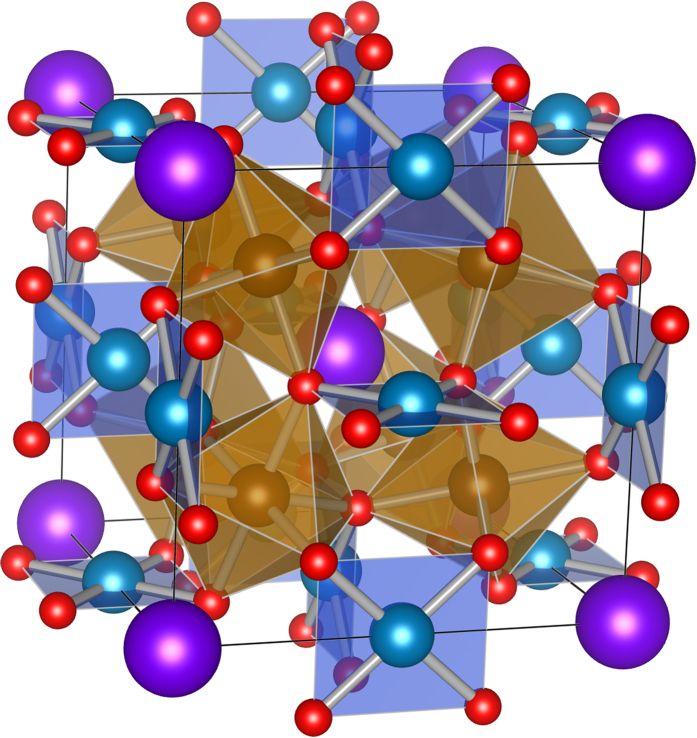
Conventional cell of EuCu_3_Fe_4_O_12_. Eu atoms are purple, Cu are blue, Fe are brown and O atoms are red colored.
